# The effects of sociocultural changes on epistemic thinking across three generations in Romania

**DOI:** 10.1371/journal.pone.0281785

**Published:** 2023-03-08

**Authors:** Amalia Ionescu, Raluca Furdui, Alin Gavreliuc, Patricia M. Greenfield, Michael Weinstock

**Affiliations:** 1 Department of Psychology, University of California, Los Angeles, CA, United States of America; 2 Department of Psychology, West University, Timisoara, Romania; 3 Department of Human Evolutionary Biology, Harvard University, Cambridge, MA, United States of America; 4 School of Education, Ben Gurion University of the Negev, Beer-Sheva, Israel; Polytechnic Institute of Viana do Castelo, PORTUGAL

## Abstract

When people experience abrupt social change, from less education to more, from less technology use to more, from a homogeneous to a heterogeneous social environment, can their epistemic thinking adapt? When divergent opinions suddenly come to be valued, does epistemic thinking shift from absolute to more relativistic? We investigate whether and how these sociocultural shifts have produced changes in epistemic thinking in Romania, a country that fell from communism and started democracy in 1989. Our 147 participants were from Timisoara and fell into three groups, each experiencing the shift at a different point in their development: (i) born in 1989 or later, experiencing capitalism and democracy throughout life (N = 51); (ii) 15- to 25-years-old in 1989 when communism fell (N = 52); (iii) 45 or older in 1989 when communism fell (N = 44). As hypothesized, absolutist thinking was less frequent and evaluativist thinking, a relativistic epistemological mode, was more frequent the earlier in life a cohort was exposed to the post-communist environment in Romania. As predicted, younger cohorts experienced greater exposure to education, social media, and international travel. Greater exposure to education and social media were significant factors in the decline of absolutist thinking and the rise of evaluativist thinking across the generations.

## Introduction

In our daily lives, we rely heavily on our ability to make sense of multiple and discrepant knowledge claims. When presented with diverging arguments regarding a particular topic, we are faced with having to make the decision regarding which view is right, or whether one view could be more right than the other. The process by which we make sense of differing knowledge claims and coordinate objective vs. subjective aspects of knowing has been termed by researchers “epistemic thinking.” Epistemic thinking refers to people’s understanding about the nature of knowledge and knowing; it has been a growing subfield in learning psychology [1]. Essentially, epistemological understanding involves an awareness of the mental states of knowing or not knowing, of being certain or mistaken, and of the role of perception, communication, and inference in providing evidence for these states of knowledge, ignorance, or belief [2].

In ecologies characterized by high levels of formal education, general models of epistemic thinking suggest a developmental progression that begins with a view that knowledge is objective and absolute, then moves to a view that knowledge is extremely subjective and uncertain, then moves to a view that knowledge has sources that are both objective and subjective and that require evaluation and interpretation [2]. These views have been given concrete terms, namely the absolutist, multiplist, and evaluativist levels of epistemological understanding [3].

The absolutist view posits that assertions are facts correct in their representation of reality. There exists the possibility of false belief, but only insofar as it is something temporary that could be remedied via examination of the external reality. While examining two divergent claims regarding the same argument, from the absolutist perspective one would say that one side is completely right and the other one not at all.

The next level of epistemological understanding, the multiplist level, posits that assertions are opinions freely chosen by and accountable only to the owners. From the multiplist perspective, one would say that both sides of an argument could be right, as it is only a matter of opinion and one can never know the truth, as knowledge is entirely generated by human minds and is uncertain.

Lastly, the evaluativist stage, which, in ecologies characterized by high levels of formal education, is considered the most mature stage in epistemological understanding, posits that assertions are judgments that can be evaluated and compared according to criteria of argument and the evidence provided. From the evaluativist perspective, one would argue that both sides of an argument could be right in theory, but, based on the evidence provided, one could be more right than the other. This concept is based on the fact that, although knowledge is generated by human minds and is uncertain, it is susceptible to evaluation. Both multiplist and evaluativist thinking constitute an awareness of knowledge being subjective and contextual; in contrast, absolutist thinking is based on an objective view of knowledge and how it is acquired.

Researchers have found that there are particular ages when humans first reach the ability to think from a multiplist or an evaluativist perspective. Developmental evidence shows that epistemological development is dependent on theory of mind, particularly children’s understanding of second-order beliefs. Second-order beliefs constitute an awareness that others possess (false) beliefs, and that they themselves also have beliefs about what others know and that these beliefs themselves might be right or wrong [4]. This does not develop until about six years of age, which is when children first develop an understanding of evidence, and thus first begin to reason using the evaluativist perspective.

However, even in fifth, eighth, and 12th grade, evaluativist thinking is shown by a minority of participants in a U.S. sample. Nevertheless, by fifth grade, multiplist thinking has become predominant, being shown across domains by a mean of 73% of fifth graders; it remains the majority mode through twelfth grade [3]. Using a similar instrument in Israel, Weinstock, Neuman, and Glassner [5] confirmed that multiplist thinking was more frequent than the other two modes within a similar age range.

In the U.S. sample, evaluativist thinking doubled from a mean of 15% across domains in fifth grade to a mean of 30.8% in twelfth grade. This pattern shows development towards evaluativist thinking. However, even in 12th grade, evaluativist thinking is still not a majority mode of thought. (These figures are based on author’s calculations from data presented in Table 4 of Kuhn, Cheney, and Weinstock [3]).

At any given developmental stage, epistemic thinking varies across domains. Kuhn and Weinstock [2] proposed that the transition from absolutist to multiplist thinking may occur much earlier in domains of personal judgments (such as taste and aesthetics) than in domains concerned with truth judgments about the physical world. In contrast, they propose that the transition to evaluativist thinking occurs much earlier in domains of truth judgments, as people recognize the possibility of evaluating theories and of using empirical evidence to do so [2]. Hence, we used multiple domains in the interview instrument used in the present study.

### Environmental influences on epistemic thinking

Researchers have found that these levels are not necessarily levels of normative cognitive development, but rather that the level of epistemic thinking one uses in a particular domain can depend on a multitude of factors, including level of knowledge of a certain topic, education, contact with a diverse environment, as well as cultural values and differences [6–10].

Advanced education is a particularly powerful factor in the development of the evaluativist mode of epistemic thinking. So far, the only group in which a majority of participants (72%) developed evaluative epistemic thinking across domains were Ph.D. candidates in educational philosophy [3]. This study, and others using different methods [6, 8, 9] also found that there is an increase in evaluativist thinking with and during undergraduate studies, and, in particular, with graduate education.

Exposure to a socioeconomically and culturally diverse environment has also been found to promote a shift toward multiplist and evaluativist thinking, both within [11] and outside of educational settings [10]. The extent to which exposure to diversity occurs within a culture depends on its social ecology, which then influences its values and socialization practices [12]. In that regard, it could be that particular practices, which vary between cultures, tend to promote epistemic development.

Studies examining the effects of cross-cultural differences on epistemological understanding and its development have found variation in the way that knowledge and knowing are understood [13, 14]. Lee [15] found that graduate students in Korea tended to believe in the dominant role of epistemic authorities (e.g., textbooks, professors, etc.) in their learning, more so than their U.S. counterparts. However, Lee [15] used Schommer’s [16] instrument in his study, which was later found to have low internal validity, and researchers deemed it inappropriate to be used cross-culturally [17].

More recently, however, Karabenick and Moosa [18] found that Omani college students were more likely to view scientific knowledge as certain and accept authority as the source of truth than U.S. college students. Similarly, when comparing Bedouin and Jewish adolescents in Israel, the Bedouins were more absolutist in their thinking, even in domains of taste and aesthetics, than the Jewish adolescents who leaned more toward multiplist and evaluativist thinking [14].

However, more recently, Weinstock [7] examined three generations in a rural Arab village in Israel. Israeli Arabs had undergone considerable social change in the last half century: a higher rate of postsecondary education and much wider use of communication technologies. As expected, these social changes were reflected in intergenerational shifts in epistemology in the village: each generation from grandmother to mother to adolescent girl became more subjectivist and less absolutist in their thinking than the generation before them. Within the sample, sociodemographic characteristics representing greater exposure to diverse people and ideas through communication technologies, as well as increasing parental education across the generations, accounted for the generational increase in subjectivist thinking. Such findings suggest that social change plays a role in the development of epistemic thinking.

The new question in this study is whether sudden sociocultural changes can affect epistemic thinking. When people experience an abrupt social change, from low levels of education to higher education, from low technology to high use of technology, from a homogeneous to a heterogeneous social environment, can their epistemic thinking adapt? Once people find themselves in an environment where divergent opinions are suddenly valued even though they were not before, does their epistemic thinking shift from absolutist to multiplist or evaluativist? The present study investigates how these specific sociocultural shifts produce changes in epistemic thinking. Based on Greenfield’s [12] theory of social change, cultural evolution, and human development and on our knowledge of social change in Romania, we expected the environmental changes to be correlated with each other.

### Studying epistemic effects of rapid sociocultural change in Romania

Because of its rapid sociocultural change, Romania provided a natural laboratory where these issues could be addressed. In the last three decades, Romania has undergone rapid economic reform as it politically shifted from communism to democracy in 1989. This reform was supported by urbanization, which is highly associated with a shift from homogeneous to heterogeneous social environments, and educational expansion, characteristic of a main trend in globalized social change [12]. Prior to 1989, during communism, people had limited exposure to a diverse environment, either within or outside of educational settings. This situation was manifest through limited exposure to technology, particularly media, and limited exposure to literature that did not directly support communist ideals. Additionally, citizens were not allowed to travel outside of the country. This type of homogeneous, limited environment should foster more absolutist thinking.

Moreover, previous studies on this area indicated that, beyond considerable changes that could be easily recognized in public life, changes in social, economic, and political environment (in terms of “liberalization”), and in behavioral everyday life register did not occur. That is, the collapse of communism was not accompanied by the same radical changes in terms of fundamental social attitudes and social values. For instance, Voicu [19], using the World Values Survey to investigate eight different generational cohorts in Romania (the oldest born between 1920–1939, the youngest born in 2000 or after) showed that most fundamental social attitudes (except for attitudes towards religion, which recorded an important decrease) are more stable than changed for these cohorts–despite the fact that exposure to socialization practices (more autocratic ones in communism and more democratic ones in post-communism) was completely different between earlier and later cohorts.

Furthermore, another study conducted on generational representative samples in Romania focused on ethical values showed that between the “parents” (60 year-old generation, mainly socialized in Communism), the “intermediate cohort” (45 year-old generation, of the “young people” in 1989, the year of Romanian revolution, who have lived the childhood and their adolescence in Communism and the mature period of life in post-Communism) and the “children” of their “parents” (30 year-old generation, who exclusively lived in post-communist) there were not any significant differences in terms of value orientation [20]. Thus, the empirical evidence could support the presence of transgenerational patterns of values and fundamental attitudes in post-communist Romania.

Although this particular societal background suggests stability rather than change in values, the current study examines the impact on epistemic thinking of different socialization practices (specifically in communism and post-communism) because of belonging to distinct generations. There is reason to think that social change may shift epistemic thinking before affecting cultural values. Among Arab Israelis in Northern Israel, epistemic shift predicted value shift, rather than vice-versa [7].

After 1989, as the country underwent a political shift, it opened its borders and later became part of the European Union in 2007, making it possible and accessible for people to travel outside of the country. In the present day, technology use is similar to that in the United States, supporting unlimited access to information and literature from global sources. Romania also underwent education expansion, with a diversification of secondary education and the introduction of alternative education models (Montessori, Waldorf, etc.), and a more selective admission to higher education [21].

According to Daniel Lerner [22] in his book titled “The Passing of Traditional Society,” which focused on social change in the Middle East, opinions develop as a result of the presence and use of media. In the Middle East at that time, it was newspapers and radios; in Romania after 1989, it was television and the Internet. Thus, after the political shift in Romania, the country broadly transformed into an environment more likely to support the existence of divergent opinions, a situation which, in turn, is associated with fostering more multiplist and evaluativist types of thinking.

Education also underwent both reform and expansion. Before 1989, the Ministry of Education set the curriculum, which was heavily influenced by communist doctrine. Teaching focused on memorization for state exams. One of the immediate educational reforms after 1989 was to rid the country of socialist ideology classes. Private schools and universities grew. Student enrollments almost doubled in just a few years; and the number of university faculties tripled [23].

Intergenerational change in epistemic thinking has been studied in the Middle East [7, 22]. The effect of the transition from communism on social development has been studied in Central and Eastern Europe [e.g., 24]. However, this is the first study on cognitive changes brought about in a formerly communist country, with its unique form of authoritarianism and collectivism.

### Is there a sensitive period for the development of epistemic thinking?

Taking a developmental perspective, we thought that the development of multiplist and evaluativist thinking, along with the reduction of absolutist thinking, might depend on the age at which one experienced the shift from communism to democracy. The concept of a sensitive period in development is basic to this hypothesis. Sensitive periods reflect developmental windows characterized by heightened neural and behavioral plasticity in response to environmental stimuli relevant to a particular capacity; following this age-related window, plasticity declines with a corresponding decline in the final level of development. Sensitive periods unfold across levels from genes to behavior [25].

The particular developmental window varies with the nature of the capacity and its age of normative development. For face discrimination, the developmental window for experiencing relevant stimuli is between six and nine months of age [26]. For learning a first language, the developmental window for experience with a particular language begins to close by four years of age [27]. For the mastery of rhythm by professional musicians, the sensitive period for musical training begins to close by seven years of age [28].

For epistemic thinking, normative developmental change has been documented from second grade through the early 30s, so long as formal education continues [3, 8]. However, holding education constant across four age groups in the U.S., Kuhn [8] found no differences in epistemic level, comparing four age groups: teens, 20s, 40s and 60s {summarized in Hofer & Pintrich [29]). This pattern indicates that age alone is not a factor in epistemic development after the teenage years—a finding suggestive of a developmental window or sensitive period for epistemic development up through high school in a society with universal education through 12^th^ grade.

In this study, we wished to explore more definitively, through a natural experiment, the possibility that there might be a sensitive period for the development of epistemic thinking. In such a case exposure to the environmental changes instigated by the fall of Communism after one’s early 30s would stimulate less epistemic development than exposure earlier in life. In order to test this sensitive period hypothesis, our research design compared three cohorts of Romanian participants; each cohort was first exposed to the democratic environment at a different age period. The two youngest age groups experienced democracy and a market economy during the period of normative epistemic development; the oldest age group experienced the shift later in life. The focus of this study was primarily on urban communities, as urban areas have experienced stronger effects of the sociocultural changes brought about by the political shift in comparison to rural areas.

### Quantitative hypotheses

This background and reasoning led to the following hypotheses:

Ia. Because of exposure to the post-communist environment at younger ages, younger cohorts would report higher levels of education, more current use of social media, and more frequent international travel than older cohorts.
Ib. Level of education, social media use, and international travel would be intercorrelated with each other, given that all are part of the development of democracy and a commercial economy [30].
ii. Sensitive period hypothesis: We predicted a sensitive developmental period for responding to more educational opportunity, communication technology, and travel possibilities with epistemological change. Specifically, we predicted an age gradient such that the greatest effect of the ecologies of democracy and a market economy in producing less absolutist and more relativistic epistemological perspectives would be for cohorts who experienced this ecology earlier in life. We expected participants from the youngest cohorts (exposed to the post-communist environment during normative epistemic development) to show more multiplist and evaluativist tendencies than the oldest cohort. In contrast, we expected participants from the oldest cohort (first exposed to the post-communist environment after age 45) to show the most absolutist tendencies. We expected the middle cohort (first exposed to the post-communist environment between age 15 and 25) to be somewhere in between in both forms of reasoning.
(iii) We predicted that there would be associations between epistemic patterns and participants’ level of education, amount of international travel, and general exposure to different opinions, either within or outside of educational settings, as well as age. Namely, it was expected that higher levels of education, international travel, and general exposure to different opinions through social media would be associated with fewer absolutist and more multiplist and evaluativist responses to the epistemic dilemmas and that these experiences would be more frequent among participants who went through the fall of Communism at a younger age.

### Using qualitative data to explore the experience and meaning of social change

We planned to use qualitative analysis to identify experiential mechanisms behind the quantitative results. We used qualitative data to explore the experience of social change in three areas:

1. Expanding sources of information
2. Rise of opinions
3. Generational differences in variety of opinions

We thought that expanded sources of information and exposure to different opinions in post-communist Romania would provide environmental mechanisms through which the variables of education, social media, and travel would lead to more relativistic thinking. Qualitative analysis was also used to illuminate the meaning for each generation of two variables used in the quantitative analysis: social media usage and international travel.

## Method

This study utilized a mixed-method design, generating both quantitative and qualitative data.

### Participants and procedure

Participants were recruited from Timisoara, Romania through the West University of Timisoara. The study was approved by the UCLA Institutional Review Board. The study, conducted in 2019 and 2020, focused on three age ranges: (i) 18–30 years old: participants who were born in 1989 or later and who most likely had more or less the same opportunities for traveling and exposure to the outside world as people would today. Even the oldest ones in that cohort grew up in the democratic environment virtually from birth. (ii) 45–59 years old: participants who were 15- to 25-years-old in 1989, who, because they were in their late adolescence/young adulthood during the political shift, most likely adapted to the new lifestyle and opportunities that life after 1989 offered. Most members of this group were, in fact, in the period of normative epistemic development [3, 27] when Communism fell. (iii) 75 years old and over: participants who were 45+ in 1989. Including traveling, media use, etc. This group was in the age range of participants who, based on age alone, had not shifted epistemic level after their 20s [8] and therefore were outside the expected sensitive period for the development of epistemic thinking. This group most likely had adapted less to the new lifestyle and had not taken advantage of all the opportunities life after 1989 offered.

There were 147 participants total; 51 in the youngest age group, 52 in the middle age group, and 44 in the oldest age group. All participants spent the entirety of their lives in urban areas in Romania. All participants provided verbal consent.

After giving consent, the participants were given a structured interview that consisted of three parts: an evaluation of epistemic thinking through dilemmas followed by qualitative questions, sociodemographic questions, and questions regarding social change. Although the youngest age group did not experience the political shift in 1989, it is possible that they might have experienced some changes throughout their lifetimes regardless. The questions were adapted to each age group such that they would be relevant to that particular cohort.

### Sociodemographic questions

The sociodemographic questions were asked as a means to gain information about the participants’ education level, their occupation, opportunity for interaction with diverse groups of people through travel, personal technology and media use, etc. (see S1 Appendix). They were asked questions such as “what cities/towns have you lived in throughout your life?”; “what is your level of education? (if applicable) What university did you attend?” S1 Appendix contains all the sociodemographic questions.

#### Social media variable

The variable of social media use was developed by combining responses to the questions “do you use social media” and “if yes, how frequently?” These questions yielded responses on a four-point scale: 0: no social media, 1: rarely use, 2: frequently use, 3: daily use.

#### Education variable

For education, we categorized responses under five different levels: 0: no high school; 1: high school completed; 2: college/vocational school completed; 3: Master’s/PhD/etc. completed. We also asked participants about where they attended high school or college, if applicable. We chose these levels because during piloting, we noticed that the discrepancy in education began with high school given that primary school was mandatory.

#### Travel variable

This binary variable was based on whether they reported ever traveling out of the country. It was selected for our analysis in preference to an alternative ordinal measure because it correlated with other independent and dependent variables, whereas the ordinal measure did not.

### Questions regarding social change

Given the rise of media after 1989, we asked questions intended as a means to gain information about the changes that participants experienced/observed throughout their lives (see S1 Appendix). The middle and oldest generation were asked questions that pertained specifically to their experiences during the transition from communism to a democracy, and the youngest generation was asked questions about any changes they might have experienced throughout their lifetimes. The questions served as a starting point for the qualitative interview, and some of the participants were given spontaneous follow-up questions based on the answers that they provided to the questions in S1 Appendix. However, most participants provided responses strictly to the questions that were pre-set in the interview.

### Epistemic dilemmas

The participants were presented with 10 epistemic dilemmas adapted from Kuhn, Cheney and Weinstock [3] concerning judgments of personal taste, aesthetic judgments, value judgments, judgments of truth about the social world, and judgments of truth about the physical world. Each category had two dilemmas; ten dilemmas total. Participants were presented with one dilemma at a time such as *“Alex says warm summer days are nicest*. *Luke says cool autumn days are nicest*.*”* Afterwards, they were asked “Is Alex right or is Luke right? Or are both of them right?” If they responded with Alex/Luke, then they were asked “Why do you think Alex/Luke is right?” If they responded with “Both of them are right” then they were asked “Could one of them be more right than the other?” and regardless of the answer, they were asked “Why?” The wording of the dilemmas, as well as the names used as examples, were changed by the authors to ensure that they are culturally relevant to the participants. All the dilemmas are presented in S2 Appendix.

### Analyses

The responses to the dilemmas were coded for absolutist, multiplist, and evaluativist tendencies based on the participants’ choices of the response options (e.g., Person 1, Person 2, both, one more so than the other) and their qualitative responses to the “why” questions, in case their explanation was in contrast with their choice. However, this conflict between choice and explanation never occurred. The responses were coded independently by two researchers who had been or are currently immersed in Romanian language and culture in order to assure interrater reliability. There was 100% agreement between the coders.

The answers which indicated certainty about one side being right and the other not at all, were categorized as "absolutist.” An example of an absolutist response given by a participant (82-years-old) belonging to the oldest age group was “Sebastian is right because I hate lying”.

The answers that suggested that correct judgment belonged to both characters were coded as "multiplist" if the arguments supported the subjectivity of opinions. An example of a multiplist response by a participant (44-years-old) in the middle age group was “They can both be right, it depends… and how and when the lie is told.”

Finally, in the situation where the participants emphasized the graduated nature of truth, yet determined that one of the characters was more right than the other, depending on several factors (e.g., scientific support, context), the answer was categorized as "evaluativist.” An example of an evaluativist response by a participant (20-years-old) belonging to the youngest age group is “They can both be right. Yes, again, one might be more right than the other, but all depends on the book they have, how scientifically accurate it is.” Further examples can be found in the supplemental materials.

We then conducted quantitative analyses of the distribution of types of epistemic response in each generation and how they related to sociodemographic information. First, we conducted Pearson correlational tests to examine possible correlations between each of our variables. Then, we conducted a multivariate analysis of variance (MANOVA) to examine differences in education, social media, and international travel between the three generations. Additionally, we conducted a two-way ANOVA to test for potential differences in absolutist, multiplist, and evaluativist responses across the three age groups. Finally, we carried out a hierarchical regression to test the effects of each of our sociodemographic variables on absolutist and evaluativist responses. All quantitative analyses were carried out in SPSS.

In contrast, we treated responses to the questions concerning the experience of social change qualitatively. The participant responses to the qualitative questions were generally thematically homogenous, with a focus on the contrast between how much access to new sources of information has expanded since the fall of communism (for the middle and oldest generations), and even in the recent years (including and especially for the youngest generation). A few responses to each of the qualitative questions are described in the results section as examples of the type of general response that was found across all participants of different ages. Some qualitative responses are included in the quantitative results section to elucidate the meaning of a particular quantitative result. Qualitative responses concerning expanded sources of information, the rise of opinions, and generational differences in the volume of opinions are presented in a separate section on qualitative findings.

The internal consistency of the ten dilemmas, calculated by *Cronbach’s Alpha coefficient*, produces a value equal to .77. That score represents a good level of item intercorrelation. Looking at each cohort separately, good internal consistency is maintained for the 18–29 age group, with a *Cronbach’s Alpha* equal to .79; the other two age cohorts have acceptable internal consistency: 45–59 years (.68) and over 75 years (.68).

## Results: Quantitative analysis

Table 1 shows the intercorrelations among all the variables used in our analyses.

**Table 1:** Intercorrelations of independent and dependent variables.

		Independent variables			Dependent variables		
	Cohort	Education	Social media use	Travel outside Romania (y/n)	Absolutist responses	Multiplist responses	Evaluativist responses
Cohort	1	.620**	- .788**	-.180*	.317**	-.097	-.320**
Education.		1	.606**	.208*	-.192*	.028	.233**
Soc. Media. use			1	.247**	-.316**	.112	.292**
Travel outside Romania				1	-.084	.087	-.001
Absolutist responses					1	-.739**	-.379**
Multiplist responses						1	-.339**
Evaluativist responses							1

*Note*. N = 147 for all correlations.

*p < .05

**p < .01.

### Hypothesis 1a. Younger cohorts would experience greater exposure to education, technology, and international travel than older cohorts

As predicted, generational cohort was negatively correlated with level of education, with participants who were older at the transition to democracy reporting a lower education level than those who were younger or not yet born (Table 1).

As predicted, technology was also negatively correlated with age, with participants who were older at the transition to democracy reporting using social media less than those who were younger or not yet born (Table 1).

When asked about their technology use, participants in the youngest and middle generations reported heavy use of technological devices such as computers, cell phones, laptops, television, iPads, etc., whereas the oldest generation largely reported only using television, radio, and phones, but no Internet. Similarly, the youngest and middle generations reported a daily use of social media (Facebook, Instagram, Snapchat, Whatsapp, etc.), whereas the oldest generation generally reported on using little to no social media.

Participants across all age groups reported traveling outside of Romania, although, as predicted, having at least one experience of international travel was also negatively correlated with age at the transition to democracy (Table 1).

Participants generally reported that the reasons for traveling were for tourism, with some participants reporting that they also traveled for business on several occasions. The youngest and middle generation participants reported having interacted with foreigners on these trips, while the oldest generation in large part reported having traveled with a large group of Romanian tourists in organized trips.

A multivariate analysis of variance (MANOVA) was carried out to see where the significant generational breaks in the experience of education, social media use and international travel lay. Generational cohort functioned as the independent variable; level of education, social media use, and international travel functioned as the dependent variables in this analysis. The overall effect of generational cohort on the group of variables was highly significant (Wilk’s Lambda, *df* = 6, 284, *F* = 49.32, *p <* .*001*, partial eta squared = .510, a large effect size). The Tukey post-hoc test indicated that for education and social media use, significant breaks occurred between every generation, with each generational cohort having, on average, a higher level of education and more frequent social media use than the one preceding it. For international travel, the Tukey test identified no significant differences among any of the three generational cohorts. However, the qualitative analysis, presented later, indicates that the nature of international travel was quite different in the oldest cohort.

### Hypothesis 1b. Level of education, social media use, and international travel would be intercorrelated with each other

As predicted, these sociodemographic variables were all significantly intercorrelated (Table 1).

### Hypothesis 2. Sensitive period hypothesis: We predicted an age gradient reflecting a sensitive period earlier in development for responding to the fall of Communism with a less absolutist and more relativistic epistemological perspective

Table 2 presents the distribution of modes of epistemic thinking across the three generational cohorts. We see that the multiplist mode of epistemic thinking is frequent in all three cohorts. It dominates in the two younger cohorts who respond, on average, to about five out of ten dilemmas in the multiplist mode; however, the absolutist mode is slightly more frequent than the multiplist in the cohort who experienced the transition to democracy in middle or old age. We also see that the evaluativist mode is least frequent in all three generational cohorts.

**Table 2:** Frequency of epistemic modes across ten dilemmas in three Romanian cohorts.

Cohort		Absolutist	Multiplist	Evaluativist
Oldest	Mean	5.18 ^a^	4.64 ^a^	0.18 ^a^
(N = 44)	*SD*	2.25	2.26	0.58
Middle	Mean	3.62^b^	5.42 ^a^	0.94 ^b^
(N = 52)	*SD*	1.88	2.18	1.65
Youngest	Mean	3.39^b^	5.20^a^	1.45^b^
(N = 51)	*SD*	2.18	2.15	1.88

*Note*. Different superscripts in the same column indicate statistically significant differences.

Table 2 shows that, as predicted, the frequency of absolutist thinking decreases in every cohort from the oldest to the youngest. In contrast, evaluativist thinking increases in every cohort from oldest to youngest. Analyses of variance (ANOVA) indicated that these generational trends were statistically significant.

There was a significant generational shift among the three generations for absolutist responses to the epistemic dilemmas (F(2,144) = 9.945, p < .001). A posthoc Tukey test showed that, on average, the oldest cohort gave a significantly higher number of absolutist responses than the two younger cohorts, who did not differ from each other (See Table 2 for means and standard deviations).

Also as predicted, ANOVA showed that the frequency of evaluativist responses differed significantly among the three generational cohorts (F(2,144) = 8.347, p < .001). A posthoc Tukey test revealed that oldest cohort gave significantly fewer evaluativist responses compared with the youngest and middle cohorts, who did not differ from each other.

Contrary to the hypothesis, analysis of variance revealed no significant difference among the three cohorts for multiplist thinking (F(2,144) = 1.590, *p* = .208). The frequency of multiplist thinking was high in all three cohorts (See Table 2 for means and standard deviations).

The significant correlations in Table 1 between cohort and absolutist responses and between cohort and evaluativist responses provide another way to document how age at the transition to democracy relates to significant shifts in epistemic thinking.

However, important questions remain: What factors are producing these generational differences in epistemic thinking? To what extent are environmental factors operative and which ones? How does the age at which the new environment was first experienced interplay with specific environmental factors: education level, international travel experience, and social media use? These questions could be addressed through regression analysis; we report these analyses in the next section.

### Hypothesis 3. Higher levels of education, international travel, and general exposure to different opinions through social media at a younger age would be associated with fewer absolutist and more relativistic (multiplist and evaluativist) responses to the epistemic dilemmas

To test this hypothesis, we turned to hierarchical regression. Regressions were carried out to determine the strength of various influences on absolutist and evaluativist thinking. Because there was no correlation of any of the sociodemographic variables or age cohort with multiplist thinking, we did not carry out a regression analysis for multiplist responses.

Correlations of under .70 among all the variables used in the regressions indicated that collinearity was not a problem. More than 20 participants per variable met the sample-size criterion for hierarchical linear regression [31]. Although the dependent variables of absolutist and evaluativist thinking deviated significantly from the normality assumption, the most recent thinking is that “linear regression…models are generally robust to violations of the normality assumption” [32, pp. 2588–2589].

We did separate regression analyses for absolutist and evaluativist thinking as dependent variables. For environmental variables we explored the three independent variables in Table 1 that correlated with the dependent variables: cohort, education level, and frequency of social media use. The absence of a correlation with the dependent variables indicated that there was no support for international travel as a predictor, and so it was not included in the regression.

For cohort, given that our analyses of variance had shown a meaningful break between the oldest cohort and the other two, we explored whether more variance could be explained by including cohort in the regression as a three-level variable or as a dummy variable in which the oldest cohort was compared against the other two.

For the variable of absolutist thinking, comparing the oldest cohort with the two younger ones explained more of the variance than making age a three-step variable. In order to see whether going through Romania’s transition after age 45 contributed anything to absolutist thinking beyond the effects of lower levels of education and social media use, the cohort variable was entered last in the regression equation. Because education was already in the past for all participants, while social media use was current, we entered education into the equation (and therefore controlled for it) before the effect of social media use was assessed. The results are shown in Table 3.

**Table 3:** Hierarchical regression: Predictors of absolutist thinking.

	Predictors of absolutist thinking		
	Model 1	Model 2	Model 3
Education level	-.19*	.00	.03
Social media use		-.32*	.09
Cohort^a^			.28^+^
*F* total	5.53*	7.99***^ ^	6.61***
*R* ^*2*^	.04	.10	.12
Δ*F*		10.11**	3.56^+^
Δ*R*^*2*^		.06	.02

^a^ Cohort = oldest generation v. other two generations.

^+^*p* = .061

*p < .05

**p < .01

***p < .001.

Each of the variables in the regression model shown in Table 3 accounts for variance in absolutist thinking. Lower educational level is correlated with higher levels of absolutist thinking (Table 1) and accounts for 4% of the variance in absolutist thinking (Table 3, Model 1). Lower social media use is correlated with higher levels of absolutist thinking (Table 1) and accounts for an additional 6% of variance (Table 3, Model 2); less social media use is the strongest predictor of absolutist thinking. Being in the oldest cohort during the transition to democracy predicts higher levels of absolutist thinking (Table 1) and accounts for an additional 2% of variance (Table 3). Cohort—i.e., age at experiencing the transition to democracy and a market economy—produces a weaker effect than the environmental variables of education and social media use (Table 3, Model 3).

Each variable accounts for a significant amount of variance, except that age is of borderline significance. The overall model accounts for 12.2% of the variance and is statistically significant (*F*(1,143) = 6.61, *p* < .001).

For the variable of evaluativist thinking, making cohort a three-step variable explained more of the variance than binary splits using dummy variables. In order to see whether the age at which one first experienced Romania as a democratic nation contributed anything to evaluativist thinking beyond the effects of higher levels of education and social media use, the cohort variable was again entered last in the regression equation. As before, we first entered education into the equation (and therefore controlled for it) before assessing the effect of social media use. The results are shown in Table 4.

**Table 4:** Hierarchical regression: Predictors of evaluativist thinking.

	Predictors of evaluativist thinking		
	Model 1	Model 2	Model 3
Education level	.23*	.09	.04
Social media use		.24*	.09
Cohort			-.23^+^
*F* total	8.29**	7.12***^ ^	5.73***
*R* ^*2*^	.05	.09	.11
Δ*F*		5.67*	2.79^+^
Δ*R*^*2*^		.04	.02

^+^*p* = .097

*p < .05

**p < .01

***p < .001.

Each of the variables in the regression model shown in Table 4 accounts for variance in evaluativist thinking. Higher educational level predicts higher levels of evaluativist thinking (Table 1) and accounts for 5% of the variance in absolutist thinking (*p =* .005) (Table 4, Model 1); it is the strongest predictor. Higher social media use is also correlated with higher levels of evaluativist thinking (Table 1) and accounts for an additional 4% of variance (Table 4, Model 2). The younger participants were during the transition to democracy, the higher their level of evaluativist thinking (Table 1); age at transition accounts for an additional 2% of variance (Table 4, Model 3). Each variable accounts for a significant amount of variance, except that age is again of borderline significance. The overall model accounts for 11% of the variance and is statistically significant (*F*(1,143) = 5.73, *p* < .001).

### Summary of quantitative results

Lower levels of both formal education and social media use predicted higher levels of absolutism and lower levels of evaluativism. Experiencing the transition to democracy in middle age (oldest generation) rather than at an earlier period of life (middle or youngest generation) predicted greater absolutism. Additionally, less use of social media was the strongest predictor of high absolutism. For evaluativism, belonging to the youngest generation was associated with a higher level of evaluativism. This generation also had the highest level of education, which was the strongest predictor of greater evaluativism.

## Qualitative analysis: The experience of social change

### Expanding sources of information

When asked about how sources of information have changed throughout their lifetimes, the middle and oldest generation reflected primarily on the difference in being able to access information that was previously prohibited during communism. Participants belonging to the oldest generation reported heavily on how during communism, they had no access to global or even domestic news due to the unavailability of information in the media: “…During communism, no one would tell us anything…;” “Before [the revolution] we were constrained, now we are free…”

The middle generation gave similar responses in regards to the change in being able to access sources of information, mentioning the change in censorship: “…[During communism,] there was nothing on television except news about Ceausescu and what his regime had accomplished…” Participants in the middle generation also heavily contrasted the use of books for entertainment during communism and the shift toward the internet and media for entertainment afterwards: “…Our source of fun was reading books by classic authors, because those were the only books that passed the censorship test… but even those were difficult to find in bookstores due to the limited amount that they would sell…” Additionally, the middle generation participants generally put emphasis on the way that this literature enhanced their “culture” and broadened their perspectives, while national television news channels along the years have done little in that regard: “…what is shown on television, especially on news channels, is still very much intended on manipulating viewers and providing them only one perspective…”

The youngest generation, though they have not experienced the political transition directly, still reported changes in sources of information throughout their lifetimes from relying solely on books and newspapers for information to also relying on the internet. This was to be expected given the technological advances made throughout the last three decades in Romania and many other countries in the world.

### Rise of opinions

Additionally, we conducted a qualitative analysis of the questions regarding the rise of opinions across generations. When asked about how frequently they find themselves having different opinions than others in their daily lives and how that has changes throughout their lives, all participants reported that they noticed divergent opinions now more than in the previous years, especially before communism. The oldest generation specifically reported that they rarely encountered different opinions in their daily lives during communism, even on mundane topics, primarily because they were afraid of accidentally mentioning something that might be punishable by the regime: “…now everyone feels free to express their opinions, but during communism no one knew what you thought about anything because you did not know who to trust or what their intentions were…”

Although not having experienced communism throughout their lives, the youngest generation also reported that the volume of opinions that they encounter in their daily lives has increased, but primarily due to the rise of social media, where people have a platform to express their opinions freely. Additionally, the majority of participants belonging to the youngest and middle generations reported that the opinions they encounter in their daily lives now, as opposed to earlier in their lives, are more superficial and not backed by evidence: “…now everyone expresses their opinions on Facebook, and everyone believes everything without seeing any evidence for it or doing any research about it…”

### Generational differences in volume of opinions

When asked whether they have noticed a difference in the volume of opinions between generations, participants across all age groups mentioned that they noticed that the younger generations seem to have more opinions about different matters than the older generations. Many of them attributed this to the oldest generation having less access to information as opposed to the middle and youngest generations. As reported by a participant from the youngest age group: “… my generation looks for information that they do not agree with, while my grandparents’ generation has less access to information and settle for what they hear on television…” Similarly, a participant from the middle generation noted that “… people from older generations are the most vehement about their opinions…” These findings suggest that although middle and youngest age group participants reported that the oldest generation seems to have expressed fewer opinions, they are inflexible regarding their opinions.

Additionally, half of the participants from the oldest generation noted that the lack of opinions that they encountered during communism was due to fear of expressing them and receiving punishment from the regime, rather than due to the fact that they did not have opinions. Altogether, these observations coincide with the responses to the epistemic dilemmas, in which the oldest generation gave, on average, the smallest amount of evaluativist responses, and the highest number of absolutist responses.

## Discussion

We have explored how the sudden sociocultural shift away from communism and towards democracy and a market economy experienced by Romania in 1989 has produced changes in epistemic thinking in Romania. We now discuss our findings in relation to each hypothesis.

### Hypothesis 1a. Younger cohorts would experience greater exposure to education, technology, and international travel than older cohorts

This hypothesis was strongly confirmed. The transition to a market economy in Romania has produced the same intergenerational increases in educational level as did a parallel ecological shift in China [33].

### Hypothesis 1b. Level of education, social media use, and international travel would be intercorrelated with each other

As in minority communities in Israel, intergenerational increases in educational level were accompanied by intergenerational increases in the use of social media as well as travel beyond the local community [34, 35]. In addition, an intergenerational increase in virtual travel—watching TV in various languages—was, in the Israeli Arab and Bedouin samples, accompanied by an intergenerational increase in education and the use of mobile technologies.

### Hypothesis 2. Sensitive period hypothesis: As new generations experience the market economy and democracy at younger ages, there will be a shift from absolutist thinking to multiplist and evaluativist thinking

The sensitive period hypothesis was confirmed also. Those participants who had experienced the transition to democracy and the market economy in middle age were significantly more absolutist and less relativistic than the cohort who experienced the transition as teens or young adults or who were born into the new society and never experienced Communism. However, note that regression analysis indicated that it was exposure to environmental influences—educational opportunity and social media—experiences that were more frequent at younger ages, than age per se.

Comparing findings with the cross-generational study of Israeli Arabs [7], we note that, in that study, generational cohort dropped out as an influence on epistemic thinking once sociodemographic mediators were taken into account. In Romania, there was a similar pattern in that generational cohort was the weakest influence on epistemic thinking; but it did not drop out entirely. The difference could be that the ecological change in Romania was much more sudden than in Arab communities in Israel, thus creating a larger generational divide.

### Hypothesis 3. Higher levels of education, international travel, and general exposure to different opinions through social media would be associated with more multiplist and evaluativist responses to the epistemic dilemmas

As hypothesized, absolutist thinking was less frequent and evaluativist thinking more frequent the earlier in life a cohort was exposed to the post-communist Romanian environment. Our sociodemographic analysis indicated that, as predicted, younger cohorts experienced greater exposure to education, social media, and international travel. Our regression analysis showed that the expansion of both education and social media across time was a significant factor in the decline of absolutist thinking and the rise of evaluativist thinking across the generations.

In parallel fashion, among Northern Arabs in Israel [7], intergenerational increases in the use of mobile technology and parental education were part of a complex of ecological variables that mediated the intergenerational decline of absolutist thinking. Earlier, Kuhn [8] had found a similar effect of education: more education was linked to evaluativist thinking, whereas less education was associated with absolutist thinking [29].

Our interpretation of the association between social media use and reduction in absolutist thinking, as well as our qualitative data, focus on the fact that social media provide exposure to multiple perspectives. Weinstock and Zviling-Beiser [10] found that another source of multiple perspectives, spending three years in the economically and culturally diverse Israeli army before university, was associated with a lower rate of absolutist thinking, compared with going straight to university without any army experience.

Although the experience of international travel increased across the generations in Romania, its presence or absence was not a significant influence on mode of epistemic thinking. However, qualitative analysis suggested that traveling out of the company of fellow Romanians might have been a significant factor; this is a topic amenable to future study.

### Implications of the findings for adaptation to the online environment

The online environment, including social media and websites, has become an inconsistent and varied carrier of information, a situation that calls for critical thinking [36, 37]. Barzilai and Zohar [38] applied modes of epistemic thinking to real-world tasks of website evaluation. The same question format utilized in the present study was used to assess Hebrew-speaking Israeli sixth-graders’ mode of epistemic use thinking as it related to website evaluation:

If two websites make opposite claims about the question“. . . .,”can only one site be right or could both be somewhat right?

If students say that only one can be right: Why? How can you tell which one is right?

If students say that both be somewhat right: Why? Could one of the websites be more right than the other, or are they both equally right?

If students say that one cannot be more right than the other: Why?

If students say that one can be more right than the other: Why? How can you tell which one is more right?

Based on responses to a series of questions in this format, children were classified as evaluativist or absolutist. Compared with absolutists, evaluativists were more likely to use website perspective or bias as a criterion when they evaluated actual websites. Searching for information on actual websites in the course of the study, evaluativists correctly identified and more often compared website point of view than did absolutists. When the researchers asked children to construct an argument from actual websites, evaluativists frequently integrated information from more than one website; in contrast, absolutists more often used a single website to construct their arguments. Last but not least, compared with absolutists, evaluativists more frequently expressed the need to take into account multiple perspectives in order to make web-based information more trustworthy. Hence, the evaluativist mode of thinking epistemic led to the kind of critical thinking that is adaptive in evaluating online information. Consequently, our findings—that increased education and increased social media use lead to a higher rate of evaluativist thinking—imply a more critical approach and better adaptation to online environments, environments that have become a central component of today’s informational ecology in Romania, as well as elsewhere in the world.

### Summary

The most important environmental mechanism for suppressing absolutist thinking was social media; our qualitative data indicate that this could be because social media entailed exposure to and expression of diverse opinions. The most important mechanism for augmenting the development of evaluativist thinking was formal education. Our qualitative data suggest that a possible causal mechanism may be the expansion of information sources in post-communist educational reform—both in school and outside of school. We can conclude that education and communication technology play a crucial role in epistemic development.

However, multiplism appears to be an exception to this generalization. Although multiplist modes of thought were dominant across the sample, contrary to hypothesis, multiplist thinking was not sensitive to either age of exposure to the post-communist environment or to post-communist opportunities for education, travel, and social media. A related pattern was found in the United States where adults of all backgrounds were likely to transition from absolutism to multiplism [3].

### Limitations and future directions

One of the potential limitations of the current paradigm pertains to the oldest generation (+75), where their interpretation of some of the items (from a semantic point of view) might differ from that of the younger generations. It is yet unclear whether the observed patterns of response are due to a specific cognitive manner of processing information based on cohort, education, and social media exposure, or were simply produced by the implicit tendency of older participants to respond to issues they did not fully understand by expressing only a single idea that would be coded by the researcher as ‘‘absolutist.” Though the dilemmas were adapted to be culturally sensitive to a Romanian population, some of the participants in the oldest generation might have had difficulty interpreting the questions due to the nature of the task. Some participants, mainly in the oldest group, did not respond to the dilemmas due to the fact that they did not understand them. These participants had to be excluded from the analysis. Thus, for future research, further adaptation should be made for the level of knowledge of the target population.

As such, in order to more carefully examine whether differences in epistemological patterns between the three age groups might be a universal experience (separate from the effects of communism directly), a future study might examine the same age ranges in urban areas in the United States, where there has been a more gradual and less abrupt sociocultural shift, particularly in regard to access to information, traveling, and exposure to diverse environments. Examining the cross-generational patterns of epistemic thinking in the U.S. might help elucidate whether there is a “sensitive period” for epistemic development, particularly by comparing the oldest generation in Romania to the oldest generation in the U.S. Given that the oldest generation in the U.S. would have experienced diverse sources of information and multiple opinions earlier in life than the oldest generation in Romania, their epistemic development may be more relativistic than in Romania. Similarly, examining cross-generational epistemic patterns in the U.S. will help us determine whether epistemic development appears to change as much across the lifespan in a culture that has experienced less abrupt social change.

## Conclusion

The purpose of this study was to explore the ways in which people across three different cohorts in Romania perceived their access to information and volume of opinions to have changed since the country has undergone a major political and sociocultural shift. Moreover, the study was intended to examine the association between these reported changes and people’s epistemic thinking. We found that the oldest generation in our sample, which experienced communism during adult life, gave the highest number of absolutist responses compared to the younger age groups. In contrast, the youngest age group gave, on average, the highest number of evaluativist responses. These patterns were most influenced by social media and formal education, suggesting that these factors might play a role in the expansion of evaluativist thinking patterns.

## Supporting information

S1 File Epistemic dilemmas example responses. Examples of participant responses to epistemic dilemmas in original Romanian and translated English.
(PDF)

S1 Appendix Sociodemographic questions. (DOCX)

S2 Appendix Epistemic dilemmas. (DOCX)
