# Supplementary material for: The effects of sociocultural changes on epistemic thinking across three generations in Romania
Source: PLoS One. 2023 Mar 8;18(3):e0281785. doi: 10.1371/journal.pone.0281785 (PMC9994674; doi:10.1371/journal.pone.0281785)
Supplement: S1 File — Examples of participant responses to epistemic dilemmas in original Romanian and translated English. (PDF) [file pone.0281785.s001.pdf]

Epistemic Dilemmas Example Responses

The categorical answers, which indicated that a character has absolute justice, were coded as "absolutist.” The answers that asserted that justice belonged to both characters were coded as "multiplist" if the arguments supported the subjectivity of opinions. In the situation where the participants keep the graduality of the truth, which depends on several factors (e.g., scientific support, context), and also note that one of the characters is more right than the other, the answer was coded as "evaluativist.”

	Absolutiste	*Pluriperspectiviste*	*Evaluativiste*
18-19 ani	„Andrei are dreptate pentru că și eu sunt de aceeași părere, și anume ca mersul pe jos cu părul ud în timpul iernii este periculos și cauzează răceală.”-R.R. 22 ani.	„Cred că variază în funcție de gusturile fiecăruia, probabil unuia îi place o aromă și celuilalt alta, depinde de fiecare. Deci amândoi au. Bineînțeles, în funcție de fiecare ce gust are. De exemplu mie îmi plac căpşunele și cuiva îi plac strugurii și na, fiecare are părerea lui.”-G.C. 20 ani	„Amândoi pot avea dreptate. Da, din nou poate avea unul mai multă dreptate decât celălalt, totul depinde de cartea pe care o au, la fel cât este de atestată științific.”-M.S. 20 ani
45-59 ani	„Alex are dreptate pentru că, tot după părerea mea, zilele de vară, zilele mai călduroase sunt mai plăcute.” A.B-56 ani	„Amândoi pot avea dreptate, depinde... și minciuna în ce formă și sub ce formă e pusă.”-D.C. 44 ani	„Pot să aibă amândoi dreptate. Habar nu am de ce se întoarce un criminal la.. și dacă se întoarce și de ce se întoarce la locul crimei. Pot, teoretic, tehnic, dacă sunt specializați să aibă dreptate sau fiecare în parte într-un anumit context. Evident. Dacă părerea e specializată și e mai bine pregătit tehnic și pe specialitate.. i-aş da ascultare specialistului.”-R.C. 46 ani
+75 ani	„Sebastian deoarece urăsc minciuna.”-A.A. 82 ani	„Acuma ce dreptate să le dau? Io la – la care să-i dau? Că fiecare are părerea lui, nu? Unu-i pare așa, altu-i pare așa, și ca și-acuma, așa le pare. Amândoi, în felu’ lor, în propria lor...preferință”-C.E.- 87 ani	„E foarte complicat, depinde de ce carte.Pot fi mai multe raspunsuri.”-S.B. 80 ani

	Absolutist	*Multiplist*	*Evaluativist*
18-19 years	”Andrei is right because I agree with the fact that walking with wet hair in winter is dangerous and causes a cold.” -R.R. 22 years.	"I think it varies according to everyone's tastes, one probably likes one flavor and the other another, it depends on each one. So they both are right.<br>Of course, depending on what everyone like. For example, I like strawberries and someone likes grapes, everyone has their own opinion. ”-G.C. 20 years	"They can both be right Yes, again one might be more right than the other, but all depends on the book they have, how scientifically accurate it is.”-M.S. 20 years
45-59 years	"Alex is right because, in my opinion, summer days, warmer days are more enjoyable."-A.B. 56 years	"They can both be right, it depends ... and how and when the lie is told."-D.C. 44 years	"They can both be right . I have no idea why a murderer returns to ... and if he returns and why he returns to the crime scene. They can, theoretically, technically, if they are specialized to be right or each one in a certain context. Obvious. If the opinion is specialized and he is better technically prepared and specialized ... I would listen to the specialist. ”-R.C. 46 years
+75 years	"Sebastian is right because I hate lying.”-A.A. 82 years	"Who can I say is right? Which one? Everyone has their own opinion, right? For one it looks like this, for another it looks like that, and like now, it looks like this. To each their own, to each their own ...preference ”-C.E. 87 years	"It's very complicated, depending on the book. There may be more answers."-S.B. 80 years
