# Supplementary material for: The effects of sociocultural changes on epistemic thinking across three generations in Romania
Source: PLoS One. 2023 Mar 8;18(3):e0281785. doi: 10.1371/journal.pone.0281785 (PMC9994674; doi:10.1371/journal.pone.0281785)
Supplement: S1 Appendix — (DOCX) [file pone.0281785.s002.docx]

**Appendix 1: Sociodemographic questions**

Participant Date of Birth: _________

1. What cities/towns have you lived in throughout your life?

Name of city/town/etc.: ___________

(*ask participant to characterize each as city, village, or suburb*)

Age when they lived there (*record the age for each place*): ___________

1. What is your occupation? *___________*
2. Did you attend high school? If so, where? Did you attend college/vocational school? If so, what college? Graduate school (Master’s, PhD)?

*-High school (if applicable):___________*

*-College/Vocational School (if applicable): ________*

*-Master’s/PhD (if applicable): ___________*

(*If none of the options are applicable, indicate highest level of schooling*): ________

Questions about traveling

1. Have you traveled out of the country? *__Yes/No__*

*…IF participant responds with “no” then skip to the questions about technology*

1. When was the first time that you traveled out of the country? ______
2. How many times have you traveled out of the country?

*- 1-5 times*

*- 5-10 times*

*- 10+ times*

*…IF participant responds with a limited number (<10 total), then follow up with question #7*

*…IF participant mentions having traveled a lot (10+ times) or yearly, then ask for how many years they have done so. Afterward, proceed to question #7.*

1. Did you travel for business or pleasure or both?
2. What countries did you visit?
3. What type of activities did you engage in while traveling?
4. Did you engage in conversation with foreigners/ attempt to immerse yourself in the culture?

Questions about technology

1. What technological devices do you use (e.g., phone, laptop/computer, iPad, TV)? ________

*IF participant indicated that they use a computer/laptop/iPad/tablet, then follow up with these questions:*

How frequently?:

- *Rarely (0-2 times per week)*
- *Frequently (3-6 times per week)*
- *Daily*

How old were you when you first started using it? _____

*IF participant indicated that they use phones, then follow up with these questions:*

What type of phone (landline, smartphone, cellphone without internet?) ________

How frequently do you use it?

- *Rarely (0-2 times per week)*
- *Frequently (3-6 times per week)*
- *Daily*

How old when you first started using it? _______

*IF participant responded with “yes” for television, then follow up with these questions:*

What do you watch on television? _______

Do you watch tv shows/movies from foreign countries/in foreign languages? If so, what countries/languages? ___________

1. Do you have access to internet in your home? ___*Yes/No*___

If so, what age did you gain access to it? ___________

1. Do you use social media? __*Yes/No*__

If yes, which platforms (e.g., Facebook, Instagram, Snapchat, Whatsapp)? _________

How frequently?

- *Rarely (0-2 times per week)*
- *Frequently (3-6 times per week)*
- *Daily*

1. Do you pay attention to the news? ___*Yes/No*___

If yes, domestic or global or both? __________

1. What sources do you use for the news (e.g., tv, social media, newspapers)? ______
2. Are any of these sources coming from outside Romania? If so, where?

Questions regarding Social Change

1. How have sources of information changed before/after 1989? (OR *“*How have sources of information changed throughout your lifetime?” for the youngest age group)
2. How often do you find yourself having different opinions than others (in your work life, at home, with your friends, etc.)?

*(if applicable – only for middle and oldest generation)*

During communism, did this occur more often, less often, or with the same frequency?

1. (*only for middle and youngest generation*) Do you see differences between your generation and **older** generations in terms of opinions (both types of opinions and volume of opinions)?
2. (*only for the middle and oldest generation*) Do you see differences between your generation and **younger** generations in terms of opinions (both types of opinions and volume of opinions)?

*(If applicable – only for middle and oldest generation):*

1. What was the biggest change that you experienced since the fall of communism?
2. What was the best thing about the political and economic shift that took place?
3. What was the worst thing about the political and economic shift that took place?
