# Supplementary material for: The effects of sociocultural changes on epistemic thinking across three generations in Romania
Source: PLoS One. 2023 Mar 8;18(3):e0281785. doi: 10.1371/journal.pone.0281785 (PMC9994674; doi:10.1371/journal.pone.0281785)
Supplement: S2 Appendix — (DOCX) [file pone.0281785.s003.docx]

**Appendix 2: Epistemic Dilemmas**

(adapted from Kuhn, Cheney, and Weinstock[3])

**Judgments of personal taste**

Alex says warm summer days are nicest. Luke says cool autumn days are nicest.

Damian says the first candy tastes better. David says that the second candy tastes better.

**Aesthetic judgments**

Stefan thinks the color red is better. Will thinks the color blue is better.

Oliver thinks the first painting they look at is better. Matthew thinks the second painting they look at is better.

**Value judgments**

Daniel has one view on whether lying is sometimes permissible. Sebastian has a different view on whether lying is sometimes permissible.

Robert thinks the government should limit the number of children families are allowed to have to keep the population from getting too big. Colin thinks families should have as many children as they choose.

**Judgments of truth about the social world**

Gabriel has one view of why criminals keep going back to crime. Dominic has a different view of why criminals keep going back to crime.

Albert thinks one book's explanation of why the Crimean wars began is right. Cesar thinks another book's explanation of why the Crimean wars began is right.

**Judgments of truth about the physical world**

Josh believes one book's explanation of how the brain works. George believes another book's explanation of how the brain works.

Andrew thinks that walking around with wet hair during wintertime causes colds. Nicholas believes that only viruses can cause colds.

**Follow-up Questions following each scenario:**

Is ___ right or is ____ right? Or could both of them be right?

If subjects respond with one of the names, then ask:

Why do you think ____ is right?

If subjects respond with “both could be right,” then ask:

Could one of them be more right than the other? Why?
